# Paraoxonase Enzyme Protects Retinal Pigment Epithelium from Chlorpyrifos Insult

**DOI:** 10.1371/journal.pone.0101380

**Published:** 2014-06-30

**Authors:** Jagan Mohan Jasna, Kannadasan Anandbabu, Subramaniam Rajesh Bharathi, Narayanasamy Angayarkanni

**Affiliations:** R.S Mehta Jain Department of Biochemistry and Cell Biology, KBIRVO Block, Vision Research Foundation, Sankara Nethralaya, Chennai, India; University of Florida, United States of America

## Abstract

Retinal pigment epithelium (RPE) provides nourishment and protection to the eye. RPE dysfunction due to oxidative stress and inflammation is one of the major reason for many of the retinal disorders. Organophosphorus pesticides are widely used in the agricultural, industrial and household activities in India. However, their effects on the eye in the context of RPE has not been studied. In this study the defense of the ARPE19 cells exposed to Chlorpyrifos (1 nM to 100 µM) in terms of the enzyme paraoxonase (PON) was studied at 24 hr and 9 days of treatment. Chlorpyrifos was found to induce oxidative stress in the ARPE19 cells as seen by significant increase in ROS and decrease in glutathione (GSH) levels without causing cell death. Tissue resident Paraoxonase 2 (PON2) mRNA expression was elevated with chlorpyrifos exposure. The three enzymatic activities of PON namely, paraoxonase (PONase), arylesterase (PON AREase) and thiolactonase (PON HCTLase) were also found to be significantly altered to detoxify and as an antioxidant defense. Among the transcription factors regulating PON2 expression, SP1 was significantly increased with chlorpyrifos exposure. PON2 expression was found to be crucial as ARPE19 cells showed a significant loss in their ability to withstand oxidative stress when the cells were subjected to chlorpyrifos after silencing PON2 expression. Treatment with N-acetyl cysteine positively regulated the PON 2 expression, thus promoting the antioxidant defense put up by the cells in response to chlorpyrifos.

## Introduction

Retinal pigment epithelium (RPE) is a monolayer of epithelial cells between the neural retina and the choriocapillaris [1]. RPE cells act as a selective barrier in regulating the movement of nutrients and solutes from the choroid to the sub-retinal space forming the outer blood-retinal barrier [2]. Loss in the RPE function is associated with oxidative stress, inflammation, fibrosis and contribute to pathophysiological processes in age-related macular degeneration (AMD), proliferative vitreoretinopathy (PVR) and proliferative diabetic retinopathy (PDR) [3]. Tumor necrosis factor alpha (TNF-α) [2], glycated-albumin [4] and oxidized low density lipoprotein [5] are capable of inducing RPE dysfunction. Pesticides like paraquat are also reported to induce oxidative damage to the RPE [6].

Organophosphate insecticide, Chlorpyrifos (CPF; O,O-diethyl-O-(3,5,6-trichloro-2-pyridyl) phosphorothioate) is common in agricultural, industrial and household pesticide formulations [7]–[8]. It is classified by WHO as class II moderately hazardous compound that has an LD50 range of 20–2000 mg//kg body weight in rat [9]. Chlorpyrifos is a neurotoxicant that inhibits neuronal and blood cholinesterase leading to overstimulation of cholinergic neurotransmission [8]. Exposure to chlorpyrifos can produce ocular toxicity with long-lasting changes in retinal physiology and anatomy [10]–[11]. Abnormal electroretinograms were noticed in rats after administration of chlorpyrifos [12]. Chlorpyrifos is reported to cause cell apoptosis, lipid peroxidation and DNA damage in mouse retina and pretreatment with antioxidants, vitamins C and E were effective in reverting these damages [13]. Chlorpyrifos is reported to induce oxidative stress by inhibiting mammalian acetylcholine esterase. In addition it also disrupts the endocrine actions of androgenic, estrogenic, thyroid and parathyroid hormones [14].

Cytochrome P450 (CYP450) metabolically activates chlorpyrifos to chlorpyrifos oxon, which is acted upon by alpha-esterases, like paraoxonase and is further converted to diethyl phosphate and 3,5,6-trichloro-2-pyridinol in the liver by the CYP450 system [15]–[16]. Chlorpyrifos is absorbed rapidly with 80% excretion in urine within 48 hr as studied in rats [17]. Paraoxonase (PON) is a calcium-dependent enzyme having enzyme activities towards varied substrates. It can hydrolyze paraoxon (PONase activity) and exhibits arylesterase (PON AREase) and thiolactonase activity (PON HCTLase). PON has 3 isoforms- PON1, PON2 and PON3 [18]–[19]. PON1 and PON3 are associated with serum HDL while PON2 is predominantly seen in tissues [20]–[21]. Antioxidant properties of human PON1 prevents oxidative modifications of lipoproteins apart from hydrolyzing oxidized phospholipids, hydroperoxides and lactones [22].

Few studies report on the detrimental effects of chlorpyrifos on retina in animal models. However, the effect of chlorpyrifos on retinal pigment epithelium has not been studied so far. This study is focused on how the RPE cells respond to the toxic pesticide chlorpyrifos *in vitro* as studied at the level of antioxidant enzyme paraoxonase.

## Materials and Methods

### Reagents

Mouse monoclonal anti-PON2 antibody (sc373981), mouse monoclonal anti-ACTIN antibody (sc32251) and goat anti-mouse horseradish peroxidase-conjugated secondary antibody (sc2005) were purchased from Santa-Cruz, USA. DMEM F12 and fetal calf serum were procured from Invitrogen (Carlsbad, CA). Dimethyl sulfoxide (DMSO), 2′,7′-dichlorodihydrofluorescein diacetate (DCFDA), ter-butyl hydroperoxide (tBH) and paraoxon (O, O-diethyl-o-p-nitro-phenylphosphate), chlorpyrifos and mithramycin was from Sigma-Aldrich (St. Louis, MO). Enhanced chemiluminescence western blotting detection reagents were from Amersham Biosciences UK, Ltd. (Little Chalfont, Buckinghamshire, UK). Redox assay kit was procured from Oxford Biomedical Research, MI, USA.

### Human RPE Cell culture

ARPE19 cells (ATCC; Manassas, VA) were grown in DMEM F12, supplemented with 10% fetal bovine serum and antibiotics (100 µg/mL penicillin/streptomycin mix) in a humidified atmosphere at 37°C with 5% CO_2_. When cells were 80 % confluent, they were shifted from DMEM F12 supplemented with 10% fetal bovine serum to DMEM F12, supplemented with 1% fetal bovine serum for 3 hr. The cells were then exposed to chlorpyrifos in 1% fetal bovine serum for 3 hr and 24 hr as for acute exposure and for 9 days in case of chronic exposure, with change of media and chlorpyrifos added at every 3 days interval. The exposure regimen was the same for all the experiments performed.

### MTT Assay

To measure cytotoxicity, ARPE19 cells were plated in a 96-well plate at a density of 5×10^4^ cells/well. After exposing the cells to chlorpyrifos for 24 hr and 9 days, medium was aspirated and 0.25 mg/ml MTT was added and incubated for 4 hr at 37 °C. The formazan crystals formed were dissolved in DMSO and absorbance at 540 nm was measured using SpectraMax M2 (Molecular Devices) with 680 nm as reference wavelength. Cell viability was defined relative to the untreated control.

### Detection of reactive oxygen species (ROS)

ROS was measured by DCFDA method. 10 µM DCFDA was added to each well and incubated for 30 minutes at 37°C and DCFDA fluorescence was measured using SpectraMax M2 (Molecular Devices) in 96-well plates at an excitation wavelength of 485 nM and an emission wavelength of 530 nM. ROS production was expressed as relative fluorescence. All assays were performed in triplicates. *N*-acetylcysteine (NAC; 5 mM), which can decrease ROS production by increasing the intracellular GSH concentration was used as antioxidant positive control, while H_2_O_2_ (1 mM) / tBH (500 µM) was used as the pro oxidant.

### Changes in Redox status

The ARPE19 cells were grown to 80% confluence in 6 well plates for the reductase assay. After treatment period, a pyridine derivative was added as a thiol-scavenger to the sample, which was then used for estimation of oxidized form of the glutathione (GSSG). Cells for GSH (without pyridine derivative) and GSSG determination were harvested in PBS. The re-suspended cells were lysed by sonication, centrifuged and the supernatant was used for the assay as per the Oxford Biomedical Kit for determination of GSH/GSSG [23]. The change in absorbance at 412 nm was measured every minute for 10 minutes.

### Determination of paraoxonase activity

The enzyme assay for estimating the paraoxon hydrolyzing activity of PON in ARPE19 cell lysates was established after modification of the existing PONase enzyme assays [24]–[26].

**PON-ase** activity was determined spectrophotometrically using 1 mM paraoxonase as the substrate and measured by increase in absorbance at 405 nm due to the formation of 4-nitrophenol for 10 min. Briefly, the activity was measured at 37°C by adding cell lysate to 300 µl of Tris-HCl buffer (100 mM at pH 8.5) containing 2 mM CaCl_2_ and 2 M NaCl. One unit is defined as 1 nmol of para-nitrophenol formed per minute.

**PON-AREase** activity was measured using 1 mM phenylacetate as the substrate. The increase in phenol liberated after hydrolysis of phenyl acetate by the addition of cell lysate was measured spectrophotometrically in kinetic mode at 217 nm following an established procedure [27]–[28]. The assay conditions were performed in buffer containing 10 mM Tris and 1 mM CaCl2, pH 8.0. One unit was defined as the enzyme quantity that disintegrates 1 µmol phenylacetate per minute.

**PON-HCTLase activity** assay was measured using γ thiobutyrolactone as substrate, and the rate of hydrolysis was measured spectrophotometrically in the kinetic mode at 450 nm (main wavelength) and 546 nm (sub wavelength) using 10 µl of cell lysate at pH 7.2, using 100 mmol/L of phosphate buffer. Enzyme activity was expressed in U/L [29].

### Semi quantitative PCR and Real time PCR for quantification of transcripts

After treatment with chlorpyrifos, the total RNA was isolated from the cells using Trizol reagent (Sigma, St. Louis, MO, USA) following manufacturer's instructions. One microgram of RNA was used for conversion into cDNA using iScript™ cDNA synthesis kit (Biorad). GAPDH was used as the house-keeping gene for normalization. Semi quantitative PCR was performed with 50 ng of cDNA. The PCR cycle conditions were: one cycle of 95°C for 2 min, 30 cycles at 95°C for 30 sec followed by 62°C for 30 sec, 72°C for 50 sec and a final extension of 72°C for 30 sec. The products were visualized under UV transilluminator on 2% agarose gel containing 0.5 mg/ml ethidium bromide. The bands obtained were quantified using ImageJ software (developed by Wayne Rasband, National Institutes of Health, Bethesda, MD; available at http://rsb.info.nih.gov/ij/index.html) after normalization to GAPDH.

10 ng of cDNA was then used for real-time PCR quantitation of products for human PON2, ARH2, JUN, NRF2, SP1, SREBP2, STAT5B with GAPDH serving as an internal control. Real-time PCR was done using POWER SYBR green PCR master mix on 7300 Real Time PCR System from Applied Biosystems. The specificity of PCR amplification products were checked by performing melting curve analysis. The primers used in the study are listed in table1.

**Table 1:** Primers used in PCR.

Gene	Sequence
arh forward	5′ CTGCCTTTCCCACAAGATGT 3′
arh reverse	5′ AGTTATCCTGGCCTCCGTTT 3′
gapdh forward	5′ GAACATCATCCCTGCCTCTACTG 3′
gapdh reverse	5′ CGCCTGCTTCACCACCTTC 3′
jun forward	5′ ACAGAGCATGACCCTGAACC 3′
jun reverse	5′ CCGTTGCTGGACTGGATTAT 3′
nrf2 forward	5′ CGGTATGCAACAGGACATTG 3′
nrf2 reverse	5′ GTTTGGCTTCTGGACTTGGA 3′
pparg forward	5′ GCCCAGGTTTGCTGAATGTG 3′
pparg reverse	5′ Tgggcgagaggcttctggca 3′
PON2 forward	5′ CCACAGCTTTGCACCAGATA 3′
PON2 reverse	5′ ATGCCATGTGGATTGAATGA 3′
PON2 forward	5′TGGGCGAGAGGCTTCTGGCA 3′
PON2 reverse	5′TGTGCCGGTCCAACAGCTGT 3′
sp1 forward	5′ GGCTACCCCTACCTCAAAGG 3′
sp1 reverse	5′ CACAACATACTGCCCACCAG 3′
srebp forward	5′ GACATCATCTGTCGGTGGTG 3′
srebp reverse	5′ GGGCTCTCTGTCACTTCCAG 3′
stat5b forward	5′ GTTGGTGGAAATGAGCTGGT 3′
stat5b reverse	5′ AGGCTCTGCAAAAGCATTGT 3′

### Silencing the expression of PON2 using siRNA

2×10^5^ cells per well were seeded in six-well plates and transfected with 10 nM of PON2 siRNA (Predesigned Flexi Tube siRNA,Qiagen) according to the manufacturer's instructions. Negative control (non silencing siRNA) incorporated in the experiment was All Stars siRNA (Qiagen) with scrambled sequence. After transfection for 24 hr, Real time PCR and western blot was done to prove the down regulation of PON2 expression. The cells post transfection with siRNA were treated with chlorpyrifos and analysed for cell viability and ROS generation.

### Immunoblot Analysis

Cells were lysed in M-PER (Thermo Fisher Scientific Inc), with protease inhibitors at 4°C and centrifuged at 5000 rpm for 10 min. The supernatant was collected and the protein was quantified using BCA method. 40 µg of protein was mixed with Laemmli sample buffer containing 100 mM DTT and electrophoresed onto a discontinuous acrylamide gel having 10% resolving gel (pH 8.8) and 4% stacking gel (pH 6.8). Gels were run on a Mini Protean III vertical electrophoresis system (BioRad) at 100V. The proteins were then transferred to Hybond-P PVDF (0.45 µ, Amersham Pharmacia Biotech) in transfer buffer (2.5 mM Tris, 19 mM glycine (pH 8.3), 20 % methanol (v/v) using a Mini Transblot cell (BioRad) at constant voltage of 100 V for each membrane for 1 hr. The non-specific protein sites on the membrane were blocked using 5% nonfat milk for 2 hr at RT on rocking shaker. The membranes were then washed thrice (3×10 min) with PBST (pH 7.4, 0.1% Tween-20). The membranes were incubated in primary antibody for 2 hr at RT. After incubation with primary antibody (1∶2000 dilution for PON2 and 1∶4000 dilution for ACTIN), the membranes were again washed for 3×10 min with PBST followed by 1 hr incubation in horseradish peroxidase conjugated secondary antibody (1∶6000 dilution) at RT. Protein bands of interest were developed using enhanced chemiluminescence system where the chemiluminescence resulting from the peroxidase-catalyzed oxidation of luminol was captured on Fluor Chem FC3 from Protein Simple. Equal protein loading was verified by immunoblotting for β ACTIN.

### Statistical Analysis

Statistical results were expressed as mean ± standard deviation of the mean obtained from each independent experiment. The results of the experimental and control groups were tested for statistical significance by a one-tailed Student's *t* test or a two-tailed ANOVA. The level of statistical significance was set at p<0.05. All experiments performed were in triplicates.

## Results

### Cell viability assay

**Figure 1:**
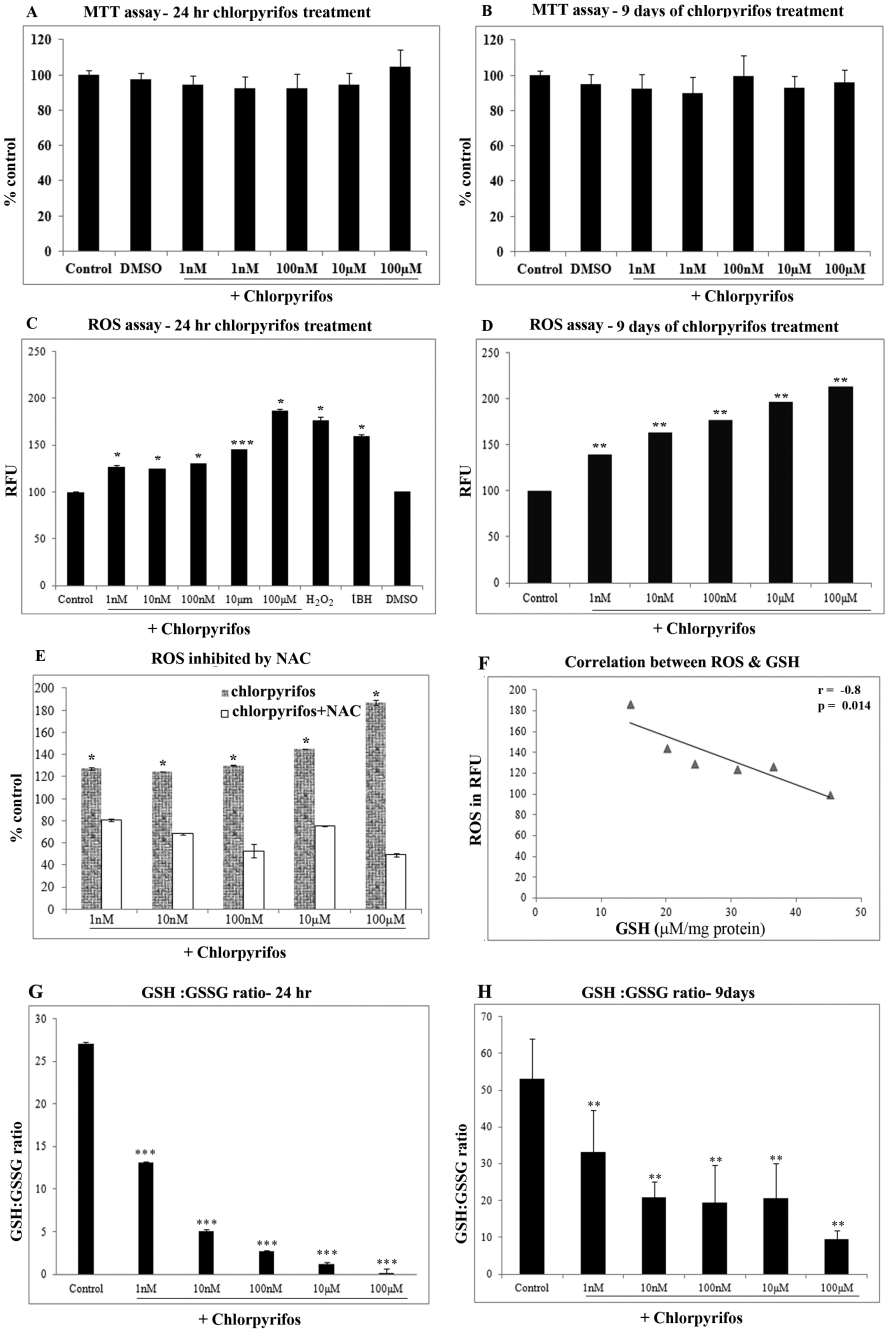
Effect of chlorpyrifos on cell viability and oxidative stress in ARPE19 cells. Effect of chlorpyrifos on cell viability in ARPE19 cells was assessed using MTT assays (A) after 24 hr chlorpyrifos treatment, (B) After 9 days of chlorpyrifos treatment. (C) ROS production measured by the DCFDA method in ARPE19 cells after 24 hr chlorpyrifos treatment. DMSO was the vehicle control used. tBH (500 µM) and H_2_O_2_ (1 mM) was the positive control for ROS generation, (D) ROS production after 9 days of chlorpyrifos treatment. (E) ROS production measured in ARPE19 cells pretreated with NAC & exposed to chlorpyrifos. (F) Negative correlation between ROS generation and the GSH level upon chlorpyrifos treatment. Dose-dependent response of the GSH/GSSG ratio to chlorpyrifos exposure (G) for 24 hr (H) after 9 days of chlorpyrifos treatment. p values are the comparison between treated control and the respective treatments. * p<0.05, ** p<0.01, *** p<0.001. All value expressed are a mean of 3 experiments done in triplicates and the values are expressed as Mean ±SD.

The ARPE19 cells were treated with varying concentration of Chlorpyrifos (1 nM to 100 µM) for 24 hr (acute) and 9 days (chronic exposure). The viability of the cells were determined by the MTT assay. The cells were viable at all the concentrations tested and there was no significant cell death even at the highest concentration of 100 µM in both acute 24 hr treatment and 9 days chronic treatment (Figure 1.A & 1.B).

### ROS generated as an index of oxidative stress

ARPE19 cells exposed to chlorpyrifos (1 nM to 100 µM) for 24 hr induced a significant increase in ROS production even at 1 nM chlorpyrifos (p<0.05) (Figure 1.C). The increase in ROS generation observed, indicates that chlorpyrifos mediates its toxic effects by causing oxidative stress to the cells. In chronic exposure conditions as well, there was a significant dose dependent increase in ROS production (p<0.01), (Figure 1.D). The maximal increase in ROS production after chlorpyrifos exposure for 24 hr was 87 %. Upon chronic exposure for 9 days there was 112 % increase in ROS seen at the maximal concentration of 100 µΜ chlorpyrifos.

### N acetyl cysteine can diminish the pesticide induced cytotoxicity

Treatment with chlorpyrifos caused an increase in ROS. Therefore, ROS scavenger namely N acetyl cysteine (NAC) was treated to see whether the cells are protected from the oxidative insult given. Pretreatment with NAC caused a significant reduction in the ROS production thus rescuing the cells of the pesticide induced oxidative stress (Figure 1E). There was 49 % decrease in ROS production when ARPE19 cells were pretreated with 5 mM NAC prior to 100 µM of chlorpyrifos exposure (p<0.05). The reduction in ROS generation with NAC pretreatment was observed in all the doses of chlorpyrifos exposure.

### Changes in levels of intracellular antioxidants

Reduced glutathione (GSH) is a major tissue antioxidant that provides reducing equivalents for the glutathione peroxidase (GPx) catalyzed reduction. When cells are exposed to increased levels of oxidative stress, the oxidized form of GSH, GSSG will accumulate and the ratio of GSH to GSSG will decrease. Determination of the GSH/GSSG ratio is a useful indicator of balance between the pro and antioxidants in cells and tissues. The level of reduced glutathione (GSH) and oxidized glutathione (GSSG) was estimated in the ARPE19 cell lysates treated with varying concentrations of chlorpyrifos for 24 hr and 9 days. When ARPE19 cells were exposed to chlorpyrifos for 24 hr, there was a significant dose dependent decrease in levels of GSH with increasing concentration of chlorpyrifos. However, there was a significant dose dependent increase in GSSG levels, the net result being that the ratio of GSH to GSSG decrease with increasing concentration of the pesticide exposure for 24 hr (p<0.001), (Figure 1G). The redox status of the cell after 9 days of chlorpyrifos exposure also showed a significant decrease (Figure 1H) (p<0.01). A significant negative correlation between the ROS generation and the GSH level was observed with chlorpyrifos treatment and is indicative of oxidative stress (p = 0.014) (Figure 1F).

### Chlorpyrifos induces the expression of paraoxonase

**Figure 2:**
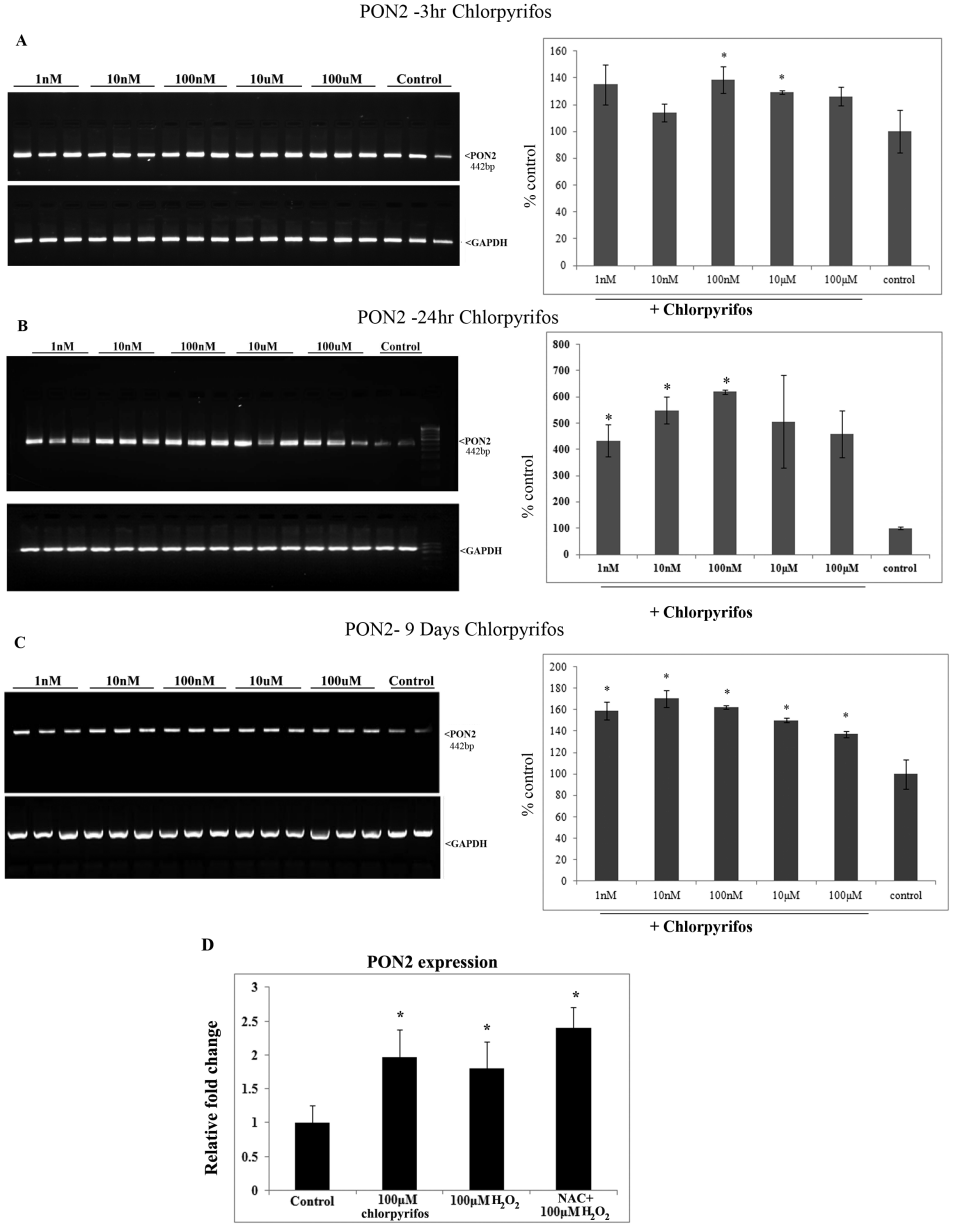
Expression of PON2 in ARPE19 cells upon chlorpyrifos exposure. (A) Expression of PON2 at 3 hr chlorpyrifos exposure. (B) PON2 at 24 hr chlorpyrifos exposure. (C) PON2 at 9 days of chlorpyrifos exposure. PCR product is visualized on ETBR gel and the Histogram represents the quantification of PCR band normalized to the GAPDH. (D) Relative quantification of PON2 expression after exposure to 100 µM chlorpyrifos and 100 µM of H_2_O_2_ with and without pretreatment with 5 mM NAC. Histogram represents the fold change after normalization to the GAPDH. All value expressed are a mean of 3 experiments done in triplicates and the values are expressed as Mean ±SD. p values are the comparison between treated control and the respective treatments.*p<0.05, t test.

Since paraoxonase is the enzyme that comes into action when an organism is exposed to organophosphate pesticides, we analyzed the expression of paraoxonase at the mRNA level in the RPE cell when exposed to chlorpyrifos. Semi quantitative PCR was employed to analyse the expression of PON2, which is the predominant tissue form of PON [21]. In ARPE19 cells also the predominant form expressed was PON2 followed by PON3 and PON1 (supplementary data). The expression of PON2 mRNA was significantly increased when ARPE19 cells were exposed to chlorpyrifos for 3 hr, 24 hr and 9 days. At 3 hr, exposure to 100 nM and above concentration of chlorpyrifos, increase in PON2 expression was observed (p<0.05). However at 24 hr, this was 6 fold higher as seen at 100 nM (p<0.05). The chronic 9 days exposure revealed an increase in the PON2 in response to all concentration of chlorpyirofs including the lowest at 1 nM. A maximum is reached at 10 nM with a 0.5 fold increase over the untreated control (p<0.05) (Figure 2). Thus, the maximal increase was seen at the end of 24 hr.

Treatment with chlorpyrifos showed a 0.9 fold increase in PON2 expression by qPCR. In order to check whether this is a ROS mediated effect, H_2_O_2_ was used as a prooxidant and the relative PON2 expression was quantified using Real time PCR. H_2_O_2_ was also found to induce expression of PON2 in ARPE19 cells. The Real Time PCR showed a significant increase in PON2 expression when 100 µM H_2_O_2_ were added to the cells (p<0.05) (Figure 2D). Pretreatment with NAC however did not abrogate the effect of H_2_O_2_ but in turn increased the PON2 expression.

### Measurement of paraoxonase enzyme activity

**Figure 3:**
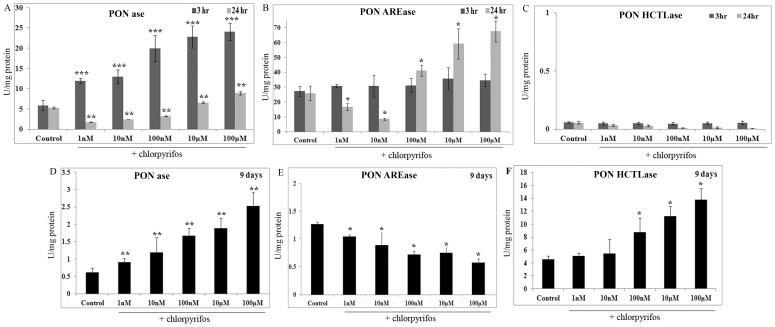
Effect of chlorpyrifos on paraoxonase enzyme activity. (A) PON-ase specific activity upon acute exposure to chlorpyrifos (3 hr and 24 hr). (B) PON-AREase specific activity upon acute exposure to chlorpyrifos (3 hr and 24 hr). (C) PON-HCTLase specific activity upon acute exposure to chlorpyrifos (3 hr and 24 hr). (D) PON-ase specific activity in chronic chlorpyrifos exposure (9 days). (E) PON-AREase specific activity in chronic chlorpyrifos exposure (9 days). (F) PON-HCTLase specific activity in chronic chlorpyrifos exposure (9 days). All value expressed are a mean of 3 experiments done in triplicates and the values are expressed as Mean ±SD. p values are the comparison between treated control and the respective treatments. *p<0.05, **p<0.01, ***<0.001.

The specific activity of PONase in the untreated ARPE19 cells was found to be 5±1.23 nmol/mg protein. The PONase activity showed a significant dose dependent increase with increase in concentration of the chlorpyrifos treatment for 3 hr (p<0.001). A maximum specific activity of 24.08±2.14 nmol/mg protein was observed at 100 µM chlorpyrifos (Figure 3A). With 24 hr treatment, the specific activity was significantly lower than the control, upto 100 nM chlorpyrifos (p<0.01). However, a dose dependent increase was observed, that was significantly higher at 10 µM and 100 µM chlorpyrifos (p<0.01). A maximum of 5 fold increase was seen at 100 µM at the end of 3 hr of exposure and less than 2 fold increase is seen at 24 hr.

With respect to PON-AREase activity, no significant change in the activity was observed after 3 hr of chlorpyrifos treatment. However at 24 hr, a decrease in specific activity was seen at lower concentration of chlorpyrifos from 1 nM to 10 nM, while at concentrations above 100 nM a dose dependent increase was observed with a maximal activity of 67±7.09 µmol/mg protein at 100 µM of chlorpyrifos exposure (p<0.05) (Figure 3B).

The PON-HCTLase activity in both chlorpyrifos treated and untreated ARPE19 cells were found to be low at 3 hr and 24 hr of chlorpyrifos exposure, with specific activity >0.1 U/mg protein (Figure 3C). However, after treatment with chlopyrifos for 9 days, there was a dose dependent increase in specific activity ranging from 4.5±0.54 U/mg protein to 13±1.76 U/mg protein (p<0.05) (Figure 3F). The specific activity of PONase and PON-AREase was found to be lower in the 9 days grown cells (control). After 9 days of chlorpyrifos exposure, a dose dependent significant increase in PONase activity was observed compared to control (Figure 3D). The specific activity of PON-AREase was found to be significantly lowered and it showed a dose dependent decrease (Figure 3E).

### Blocking PON2 expression disturbed the ARPE19 cells ability to withstand oxidative insult

**Figure 4:**
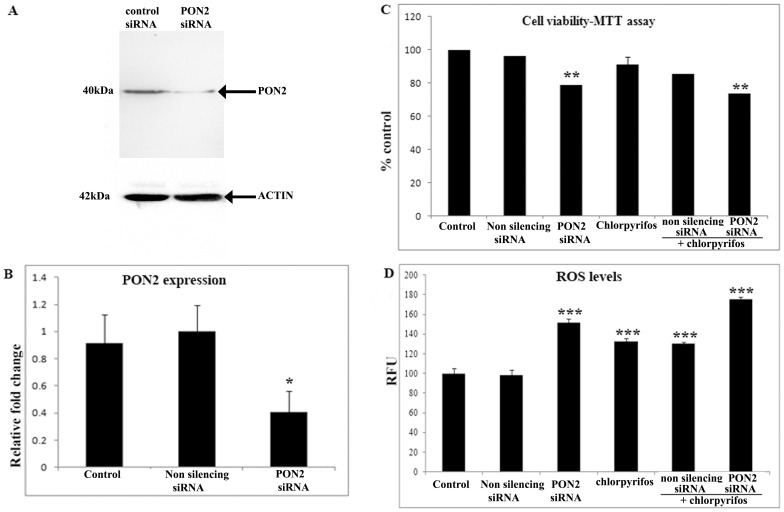
Silencing PON2 expression causes cell death and increases the ROS production. (A) Immunoblot analysis of PON2 from ARPE19 cells transfected with PON2 and non silencing siRNA. β ACTIN used for normalizing the protein load. (B) Histogram represents the expression of PON2 in the control and siRNA treated ARPE19 cells as fold change obtained from the qRT PCR. (C) Effect of 100 µM chlorpyrifos on cell viability in ARPE19 cells where PON2 expression was silenced using siRNA. (D) ROS production measured by the DCFDA method in ARPE19 cells transfected with PON2 siRNA or non silencing siRNA and exposed to chlorpyrifos for 24 hr. All value expressed are a mean of 3 experiments done in triplicates and the values are expressed as Mean ±SD. p values are the comparison between treated control and the respective treatments.*p<0.05, **p<0.01, ***p<0.001, t test.

Since PON2 expression was observed to be elevated upon exposure to chlorpyrifos study was done to determine the effect of PON2 silencing in ARPE19 cells exposed to chlorpyrifos. Down regulation of PON2 after siRNA silencing in ARPE19 cells was shown by western blot (Figure 4A) and qRT PCR (Figure 4B). Cell viability studies showed that upon silencing the PON2 expression in ARPE19 cells and exposing the cells to chlorpyrifos, there was a significant loss in viability as seen by MTT assay (Figure 4C). With 26 % cell death, a significant decrease in cell viability was observed when compared to the control cells (p<0.01). There was a 44% increase in ROS production (p<0.001) in the PON2 silenced ARPE19 cells exposed to chlorpyrifos in comparison to the non silencing siRNA (scrambled siRNA) transfected cells exposed to chlorpyrifos (Figure 4D).

### Exploring the transcription factors regulating the expression of PON by qRT-PCR

**Figure 5:**
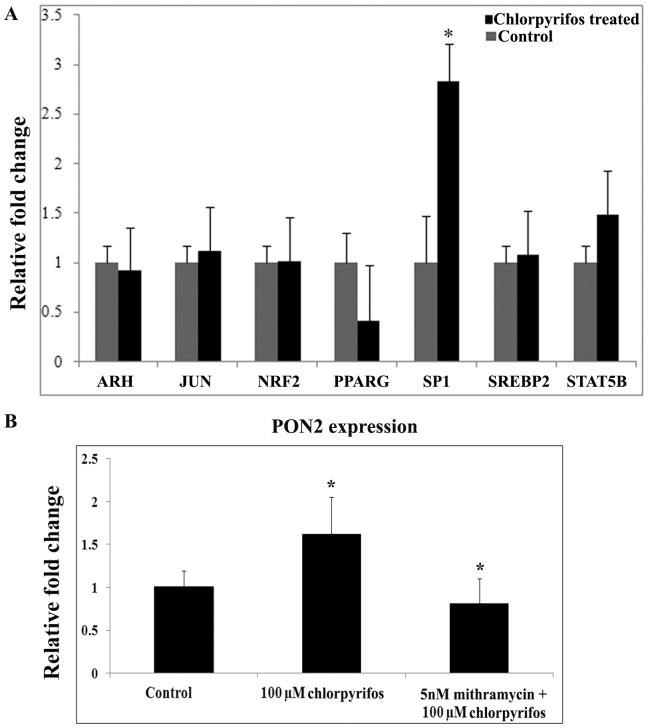
Transcriptional factors regulating PON2 expression. (A) Relative quantification of the expression of transcription factors regulating PON2 in ARPE19 cells in response to chlorpyrifos exposure for 24 hr. (B) Effect of mithramycin on PON2 expression. 5 nM of mithramycin was used to interfere with SP1 function. Histogram represents the fold change after normalization to the GAPDH. All value expressed are a mean of 3 experiments done in triplicates and the values are expressed as Mean ±SD. p values are the comparison between treated control and the respective treatments. * p<0.05.

In ARPE19 cells, chlorpyrifos increased the synthesis of PON2 and experiments where PON2 was silenced also proved that PON2 in the cells provide protection during stress. The expression of transcription factors that regulate transcription of PON2 was further looked into. The transcription factors studied were shortlisted based on literature on PON expression. Expression of transcription factors namely ARH, STAT5B [30]–[31], SREBP2 [32], NRF2 [33], JUN [34], PPARG [35] and SP1 were analysed in the chlorpyrifos exposed cells and the control ARPE19 cells. At 24 hr of chlorpyrifos exposure, there was a significant 2.5 fold increase in expression of SP1 in the chlorpyrifos exposed ARPE19 cells compared to the untreated controls (p<0.05) (Figure 5A).

To further prove SP1 mediated PON2 expression, mithramycin an inhibitor that interferes with SP1 transcription factor binding was treated before chlorpyrifos exposure. Mithramycin treated cells showed 0.8 fold decrease in PON2 expression even after chlorpyrifos challenge, as per the Real Time PCR results (Figure 5B), thereby showing that the transcription factor SP1 plays a role in regulating PON2 expression.

## Discussion

Ocular damage due to organophosphate pesticides exposure was studied by the US environmental protection agency subsequent to reports from India and Japan [36]–[37]. A few of these studies have indicated damage in the eye at the level of retina. Acute dose of Fenthion in experimental rats caused increased expression of glial fibrillary acid protein (GFAP) in rat retina [38]. Macular damage suggestive of RPE defect was noticed in significant proportion of farmers using chlorpyrifos [39].

In this study cultured human retinal pigment epithelium was treated with sub lethal dose of chlorpyrifos ranging from 1 nM to 100 µM, and no significant cell death was observed. Earlier reports on acute and chronic pesticide toxicity in mouse, revealed markers of oxidative stress such as oxidized lipids, lowering of antioxidant enzymes and DNA damage in the retina [13]. Our study revealed that chlorpyrifos treatment to ARPE19 cells induces a dose dependent ROS generation. The antioxidant N-acetyl cysteine was able to significantly reduce the ROS generated, thus showing that organophosphorous pesticide, chlorpyrifos causes an oxidative stress to ARPE19 cells. Chlorpyrifos also led to lowering of the redox status as measured by the ratio of GSH/ GSSG in the ARPE19 cells with significant reduction in glutathione levels. The activities of antioxidant enzymes superoxide dismutase, catalase and glutathione peroxidase are previously reported to be decreased in the retina of chlorpyrifos administrated mice [13]. Reduced glutathione levels associated with chlorpyriofs treatment is reported in rat brain [40] and recently in JEG-3 cells [41].

As seen in this study, ARPE19 cells were capable of withstanding the oxidative insult in response to the chlorpyrifos exposure with no significant cell death as depicted in the cell viability assay. Concentrations up to 1 mM did not show significant cell death at 24 hours (data not shown).

Organophosphorous pesticides like chlorpyrifos are cleaved by the enzyme paraoxonase. The paraoxonase (PON) gene cluster contains three members (PON1, PON2 and PON3), located on chromosome 7q21.3–22.1. Of the three, PON2 the tissue resident form is more expressed in ARPE19 cells with low levels of PON1 and PON3 mRNA (Supplementary Figure S1). PON2 is an intracellular form that protects cells against oxidative stress and is not associated with HDL unlike PON 1 and 3 [21]–[42]. In this study, the expression and activity of paraoxonase were determined in ARPE19 cells exposed to chlorpyrifos. An increase in the specific activity of PONase was observed at 3 hr, which can be attributed to the paraoxon substrate availability and its detoxification. PON2 mRNA levels are not increased at 3 hr, as much as seen at 24 hr. A 4-fold increase in PON2 mRNA expression was observed at 24 hr and this gene expression change can be attributed to the pro oxidant insult mediated signaling. This is supported by the fact that chlorpyrifos exposure at 24 hr, showed increased ROS, lowered glutathione and altered redox status apart from increase in the PON-AREas antioxidant activity. Silencing of PON2 increased the ROS levels and caused significant cell death, indicating the crucial role of PON2 in RPE. Thus, the acute exposure of chlorpyrifos to ARPE19 cells is taken care by the cellular paraoxonase activity and gene expression.

Nine days chronic exposure of chlorpyrifos at varying concentration showed a net increase in the mRNA levels of PON2 compared to the untreated control, but was less when compared to the 24 hr levels, indicating the lowering of defense through PON expression. Oxidative stress has been shown to decrease the mRNA expression of PON2 and over expression of PON2 to be protective, as studied in mice macrophages and HeLa cells respectively [21]–[43]. On the other hand, Shamir et al, showed that induced oxidative stress did not alter the mRNA expression of PON2 in Caco-2 cells [44]. A recent study by Chiapella et al indicates that the placental JEG-3 cells are able to attenuate the oxidative stress induced by chlorpyrios [41]. Thus, there seems to be variations in PON expression based on the tissue type. RPE cells detoxify the chlorpyrifos and show an up regulation of the PON2 expression probably as a defense in response to the chlorpyrifos induced oxidative stress.

Treatment with the proxidant such as H_2_O_2_ increased the PON expression, showing the ROS mediated gene expression of PON2. Interestingly this was not abrogated by pretreatment with antioxidant NAC. NAC treatment further increased the PON2 expression. This can be explained by the fact that NAC action is not restricted to just free radical scavenging or in improving the intracellular glutathione as cysteine precursor, but also in influencing the redox state of the cysteine residues of signaling molecules such as Raf-1, MEK and ERK. NAC-mediated signaling resulting in the activation of SP1 is reported [45]. Thus with NAC treatment, an increase in PON 2 expression was observed in the ARPE19 cells. Therefore, treatment with NAC, not only improves the redox status, but can also increase the antioxidant response through PON2 upregulation. The beneficial effects of NAC supplements in chlorpyrifos toxicity can be further looked into.

ARPE19 cells grown post confluent for 9 days showed an overall decrease in the activity of PONase and PON-AREase but the thiolactoanse activity showed an increase. Interestingly there was around 10 fold increase in the PON-HCTLase activity when compared to the untreated control at 9 days. The specific activity of PON-HCTLase increased dose dependently with chlorpyrifos treatment. This can also be due to the accumulation of substrates for the thiolactonase activity. However the range of physiological substrate for this enzyme activity is still unclear [42]–[46].

Thus it is observed that PONase activity plays an immediate role to detoxify chlorpyrifos and reduces cellular ROS that helps in the survival of the cell. The PON-AREase activity increases with time to handle the accumulating oxidized substrates. However, in spite of the augmented paraoxanase activity and expression, the oxidative stress and altered redox status still seems to be predominant, clearly showing that the cells antioxidant machinery is down. Studies in animal models, on long term chlorpyrifos exposure is required for further understanding the RPE metabolism and molecular mechanism in handling the chlorpyrifos toxicity especially with chronic exposure.

Amongst the known transcriptional regulators of PON, SP1 was significantly increased in RPE cells exposed to chlorpyrifos. SP1 is reported to act as a positive regulator of PON1 transcription, mediated through PKC, a cellular sensor of the intra cellular redox changes [47]–[48]. SP1 activation during excess ROS generation is previously reported and the same is seen in this study [49]. Thus based on this study it is inferred that chlorpyrifos that induces ROS generation and glutathione depletion, results in increased PON2 expression through SP1 activation, as a protective response. While regulation of ROS using antioxidants may prove to be beneficial in chlorpyrifos induced toxicity, the role of PON polymorphism in the pesticide users needs attention.

## Supporting Information

Figure S1 Expression of PON1, PON2 and PON3 in ARPE19 cells and HUH cells. HUH cells are used as positive control to show that primers of PON1 and PON3 are working.
(TIF)
