# Supplementary figures and images for: Paraoxonase Enzyme Protects Retinal Pigment Epithelium from Chlorpyrifos Insult
Source: PLoS One. 2014 Jun 30;9(6):e101380. doi: 10.1371/journal.pone.0101380 (PMC4076322; doi:10.1371/journal.pone.0101380)

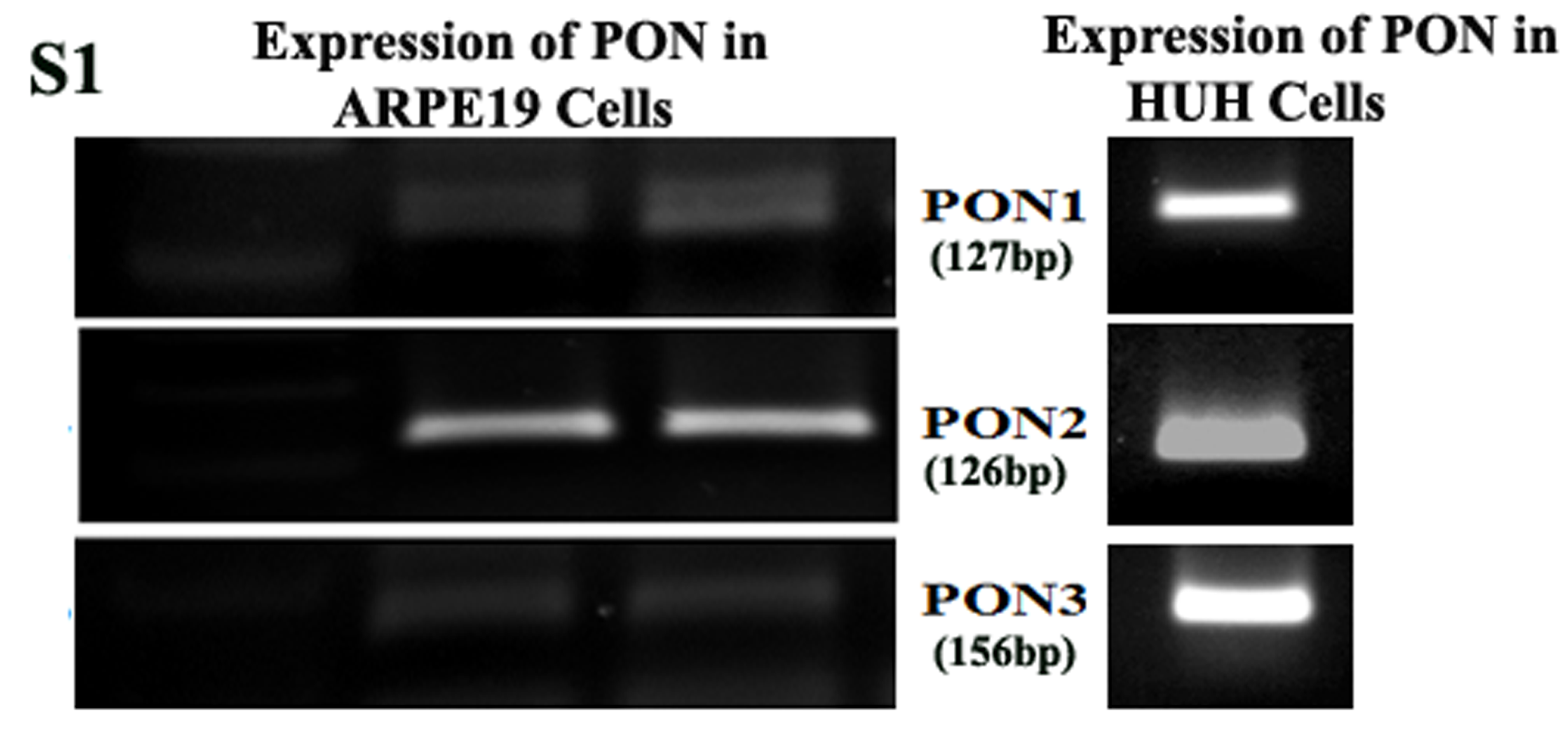

Supplement: Figure S1 — Expression of PON1, PON2 and PON3 in ARPE19 cells and HUH cells. HUH cells are used as positive control to show that primers of PON1 and PON3 are working. (TIF) [file pone.0101380.s001.tif]
